# Adsorption Behavior of a Ternary Covalent Organic Polymer Anchored with SO_3_H for Ciprofloxacin

**DOI:** 10.3390/molecules28196941

**Published:** 2023-10-05

**Authors:** Zhuoran Wang, Chuanyu Qin, Dongyu Zhao, Ziheng Wang, Dongpeng Mao

**Affiliations:** 1 Key Laboratory of Groundwater Resources and Environment of Ministry of Education, College of New Energy and Environment, Jilin University, Changchun 130021, China; qincyu@jlu.edu.cn (C.Q.); zhaody20@mails.jlu.edu.cn (D.Z.); wzh20@mails.jlu.edu.cn (Z.W.); maodp20@mails.jlu.edu.cn (D.M.); 2 Jilin Provincial Key Laboratory of Water Resources and Environment, College of New Energy and Environment, Jilin University, Changchun 130021, China; 3 National and Local Joint Engineering Laboratory for Petrochemical Contaminated Site Control and Remediation Technology, Jilin University, Changchun 130021, China

**Keywords:** covalent organic polymers, ciprofloxacin, kinetic analysis, selective adsorption

## Abstract

Owing to the poor treatment efficiency of wastewater containing fluoroquinolones (FQs), effective removal of such pollutants has become a significant issue in waste management. In this study, a ternary covalent organic polymer anchored with SO_3_H (COP-SO_3_H) was designed using the Schiff reaction and a multicomponent solvent thermal method. The synthesized COP-SO_3_H polymer possesses multiple functional binding sites, including amide groups, sulfonic groups, and aromatic frameworks, enabling it to effectively adsorb ciprofloxacin (which belongs to FQs) through mechanisms such as pore-filling effects, electrostatic interactions, hydrogen bonding, π-π electron donor–acceptor (EDA) interactions, and hydrophilic–lipophilic balance. COP-SO_3_H demonstrated outstanding adsorption performance for ciprofloxacin, exhibiting a high adsorption capacity, broad pH stability, strong resistance to ionic interference, and good regenerability. Moreover, it displayed preferential selectivity toward fluoroquinolone antibiotics. The present study not only investigates the intricate structural and functional design of COP-SO_3_H materials but also presents potential applications for the efficient adsorption of specific antibiotics.

## 1. Introduction

Fluoroquinolones (FQs) are a primary category of pharmaceutical compounds that exhibit high antibacterial efficacy and are widely used in disease treatment, infection prevention, and other areas [1,2]. Among these, ciprofloxacin (CIP), a third-generation FQ antibiotic and a prominent representative of the quinolone antibiotic class, is widely employed in livestock farming, healthcare, and aquaculture because of its cost-effectiveness, notable therapeutic benefits, and wide antibacterial coverage. Approximately 70% of these antibiotics are not metabolized and enter the environment through feces and urine [3]. CIP has been detected in diverse water bodies, including wastewater from hospitals, pharmaceutical manufacturers, residential areas, and rivers, with concentrations ranging from micrograms to milligrams per liter. Furthermore, research has indicated that the concentration of CIP in pharmaceutical factory effluents can exceed 31 mg·L^−1^, posing a significant threat to human health [4,5]. In recent years, prolonged consumption of drinking water containing CIP has led to symptoms such as anxiety, nausea, vomiting, headaches, diarrhea, and tremors [6]. High concentrations of CIP can damage the human immune system, leading to conditions such as acute renal failure, elevated liver enzyme levels, and reduced white blood cell counts [7]. Additionally, CIP inhibits the growth of photoautotrophic aquatic organisms and exhibits high toxicity at certain concentrations [8]. When exposed to residual antibiotics and their metabolic degradation products, aquatic bacteria in water environments develop resistance genes, which accelerate the development of bacterial resistance [9].

Given that CIP is continuously released into surface waters from wastewater treatment plants, agricultural runoff, aquaculture, and pharmaceutical manufacturing sites and subsequently enters the environment and threatens human health, it has become a popular research topic in the field of water environment remediation [10,11]. Various methods have been developed to remove CIP from water, including advanced oxidation processes [12], electrocoagulation [13], photodegradation [14], biodegradation [15], and adsorption [16]. Among these, the adsorption technique is distinguished by its cost-effectiveness, straightforward design, high efficiency, and compatibility with other water treatment systems. The choice of adsorbent material significantly affects the adsorption rate [17]. Thus far, inexpensive materials, such as silica dioxide, clay, zeolite, and bentonite, have been used to remove antibiotics from water. However, adsorbents commonly exhibit low porosity, small specific surface area, weak surface charge, low capacity, and poor selectivity, which ultimately diminish their removal efficiency of quinolone antibiotics [18,19]. Consequently, there has been widespread focus on designing adsorbents with exceptional extraction capabilities and significant selectivity based on the target molecular structure and physical characteristics to address these limitations [20,21].

Covalent organic polymers (COPs) are organic porous substances composed of light components, such as C, N, O, and H. The synthesis of various crystalline and amorphous COPs has been achieved, encompassing covalent organic frameworks (COFs) [22,23], covalent triazinyl frameworks (CTFs) [24], and microporous organic polymers (MOPs) [25]. Because of their strong chemical tunability, high crystallinity, adjustable pore structure, low density, and excellent stability, they are frequently employed in energy storage, gas adsorption and storage, and multiphase catalysis. Currently, sulfonated H-COF-SO_3_H exhibits selective adsorption towards paraquat and dipyridine-containing polar cations through electrostatic interactions [26]. MOPs based on the Schiff base (SNW-1) can serve as enrichment coatings for the collection of volatile fatty acids in tea and tobacco powder samples [27]. Therefore, COPs demonstrate promising adsorption performance and application prospects. However, reports on the utilization of COPs as adsorbents in antibiotic wastewater treatment are limited, particularly regarding the selective adsorption of FQs. FQ antibiotics commonly possess a molecular structure featuring two ion-binding sites, a carboxyl group, and an amine group [1]. Based on this characteristic, an adsorbent with a specific affinity towards FQs was designed to achieve efficient and selective capture of FQ antibiotics while avoiding interference from other pollutants on its adsorption performance and enhancing its overall efficiency.

To accomplish our objective, a solvothermal method was employed to prepare a porous material (COP-SO_3_H) featuring micropores and mesoporous pores. These pores serve as channels and pathways for diffusion within the adsorbent. Micropores and mesopores provide pathways for the diffusion of the adsorbate into the adsorbent material. The introduction of a sulfonic acid group and a -CO-NH- group to the adsorbent and electrostatic interactions between COP-SO_3_H and FQs are expected to enhance the selectivity for FQs and effectively remove FQs present in water. The adsorption mechanism is mainly based on the pore-filling effect, electrostatic interactions, hydrogen bonding, π-π EDA interactions, and hydrophilic–lipophilic equilibrium. To verify the adsorption mechanism, the adsorption behavior of a new covalent organic polymer, COP-SO_3_H, on FQs in water (taking CIP as an example) was examined in this study, and detailed information on the adsorption capacity, adsorption kinetics, adsorption thermodynamics, and adsorption mechanism was obtained. The present study not only investigated the selective adsorption of sulfonic acid groups and amide bonds on FQs in covalent organic polymers, but also proposed a novel approach for designing the removal of organic pollutants from water.

## 2. Results and Discussion

### 2.1. Synthesis and Characterization of COP-SO_3_H

**Figure 1:**
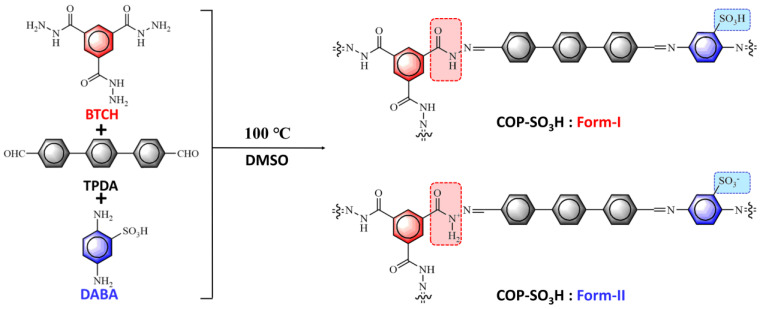
Schematic diagram of COP-SO_3_H synthesis.

**Figure 2:**
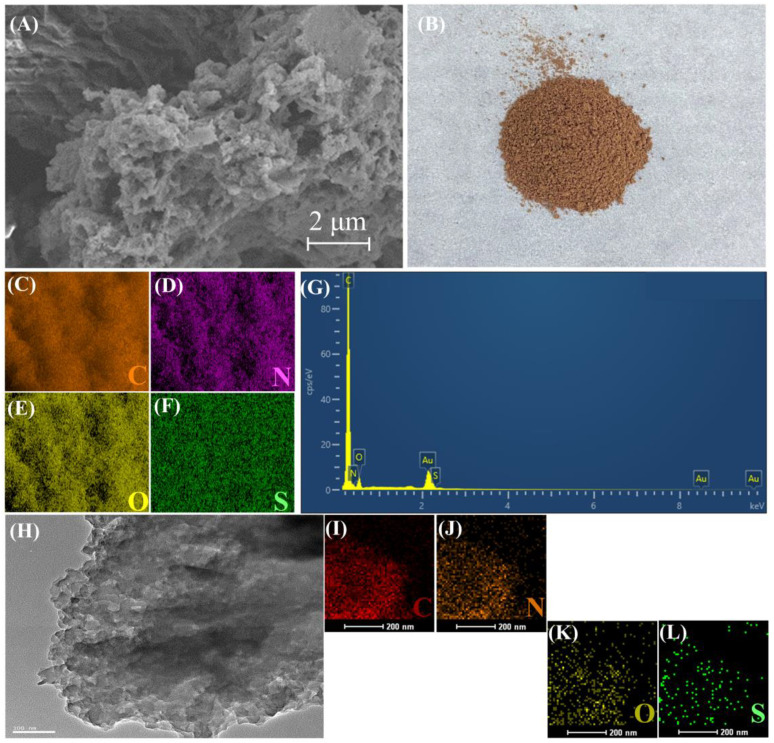
(**A**) SEM image of COP-SO_3_H. (**B**) Photograph of COP-SO_3_H. (**C**–**F**) EDX elemental mapping of C, N, O, and S. (**G**) EDS spectrum of COP-SO_3_H. (**H**) TEM image of COP-SO_3_H. (**I**–**L**) TEM elemental mapping of C, N, O, and S.

As shown in Figure 1, COP-SO_3_H was prepared by connecting BTCH, TPDA, and DABA via imine bonds, using a solvothermal method. Figure 2B shows the physical appearance of COP-SO_3_H. Figure 2A,H shows the scanning electron microscopy (SEM) and transmission electron microscopy (TEM) analyses revealing the amorphous nature of COP-SO_3_H, characterized by an internal honeycomb structure. Furthermore, elemental mapping based on energy-dispersive X-ray spectroscopy (EDX) confirmed the uniform distribution of C, N, O, and S on the surface of COP-SO_3_H, as shown in Figure 2C–F,I–L. The elemental analysis, as illustrated in Table 1, demonstrates congruence between the measured and theoretical values. Notably, the presence of S confirmed the successful anchoring of sulfonic acid groups onto COP-SO_3_H.

**Figure 3:**
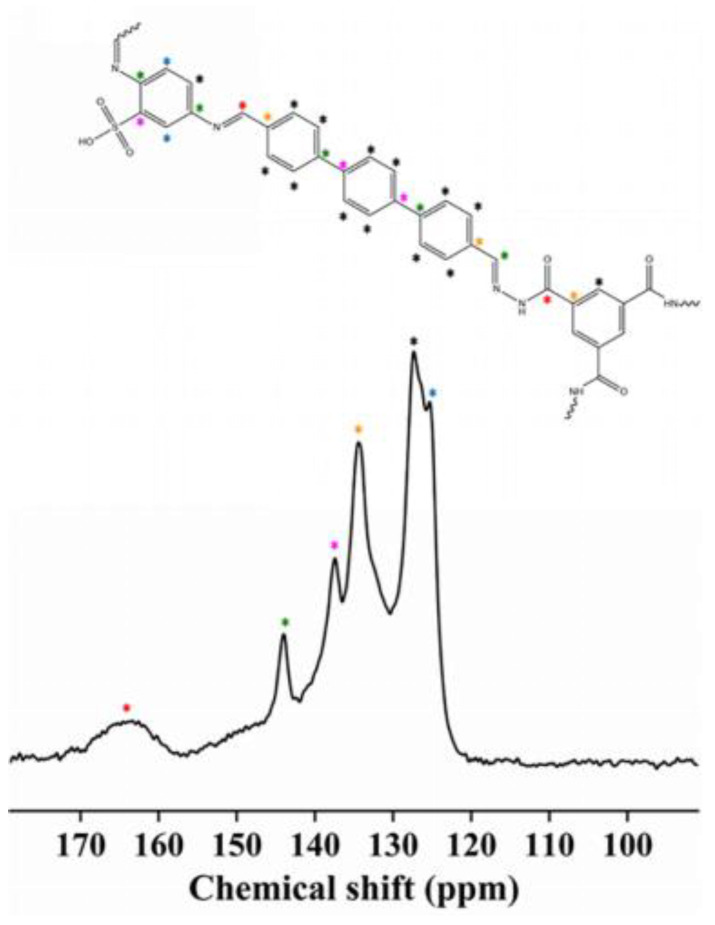
Solid-state ^13^C CP/MAS NMR spectra of COP-SO_3_H. The red stars represent carbons of imine bonds, the green stars represent carbons linked to N and benzene rings, and the others are attributed to other aromatic carbons.

**Figure 4:**
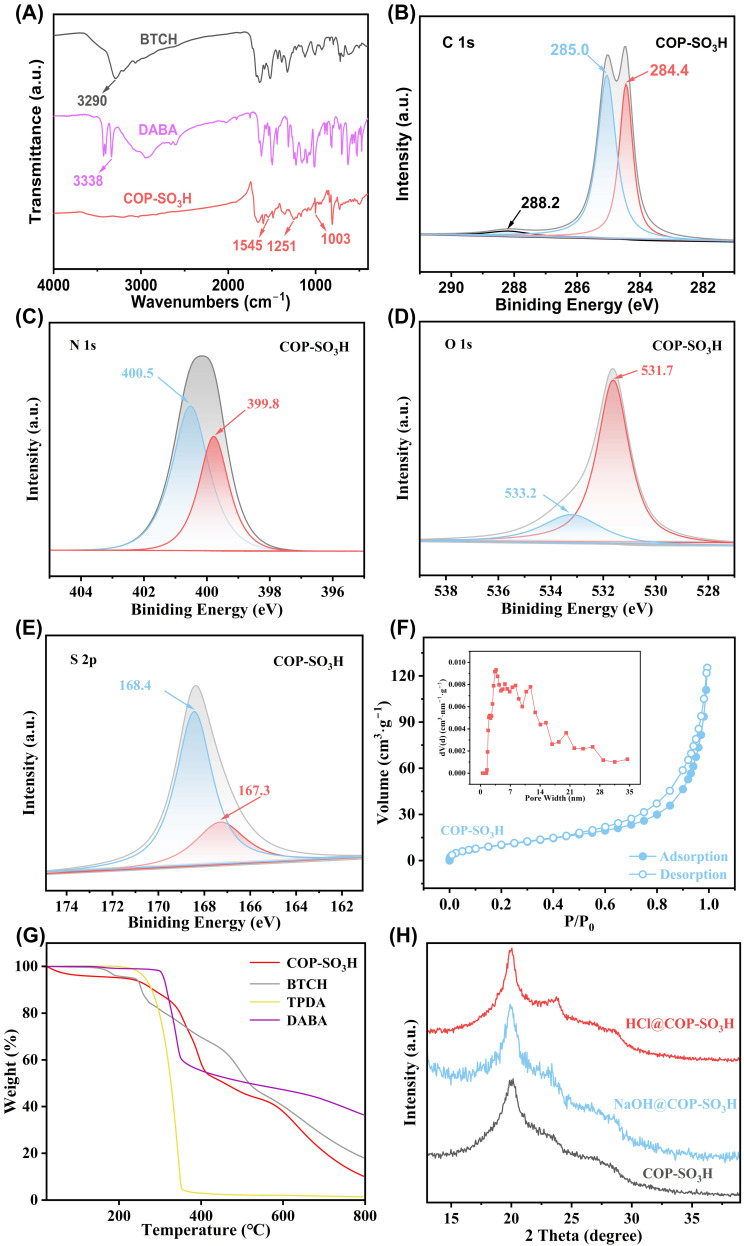
(**A**) BTCH, DABA, and COP-SO_3_H FT-IR spectra. **(B**–**E**) XPS spectra of O 1s, C 1s, N 1s, and S 2p in COP-SO_3_H. (**F**) Adsorption–desorption curves and pore size distribution of COP-SO_3_H. (**G**) TGA curve of COP-SO_3_H. (**H**) XRD spectra of COP-SO_3_H, HCl@COP-SO_3_H, and NaOH@COP-SO_3_H.

The structure of COP-SO_3_H was further investigated using 13C CP/MAS NMR (Figure 3) and FTIR (Figure 4A) characterization techniques. The FT-IR spectra exhibited characteristic peaks at 3290 and 3338 cm^−1^ for BTCH and DABA, respectively. Notably, the N–H stretching band (3290–3338 cm^−1^) was absent in COP-SO_3_H [28]. A new characteristic peak emerged at 1545 cm^−1^, attributed to imine bond formation [29], coinciding with the disappearance of the BTCH and DABA peaks, thus confirming the successful synthesis of COP-SO_3_H. Furthermore, the new peak at 1003 cm^−1^ in COP-SO_3_H indicates the stretching band of the sulfonic acid groups [28]. The solid-state ^13^C NMR spectrum of COP shows peaks at 163 ppm, which can be assigned to imine carbon and amide carbon, indicating the successful synthesis of COP [30].

The chemical elements and their states in the adsorbents were investigated via XPS analysis, which confirmed the successful synthesis of COP-SO_3_H. The XPS spectra of C, N, O, and S in COP-SO_3_H (Figure 4B–E) corresponded to the expected components. For C, the 1s peaks at 288.2, 258.0, and 284.4 represent C=O, C-S/C=N, and C-C/C=C, respectively [31]. The N elements exhibit 1s peaks at 400.5 and 399.8 eV, attributed to the formation of amine bonds in COP-SO_3_H [32]. For O elements, the 1s binding energy peaks at 533.2 and 531.7 eV indicate the presence of C=O and S=O groups in COP-SO_3_H [33]. Notably, COP-SO_3_H exhibited two distinct configurations. For S, the 1s binding energy spectrum of COP-SO_3_H reveals two peaks (168.4 and 167.3 eV), corresponding to sulfonic acid groups (SO_3_H) and sulfonate ions (SO_3_^−^). The 1s peak at 400.5 eV for N originates from the protonated secondary amine (-NH^2+^) within the Form-II structure [28].

Nitrogen adsorption–desorption tests (Figure 4F) were conducted at 77 K to determine the specific surface area of COP-SO_3_H. The BET isotherm exhibits a typical type-IV shape [34]. The BET surface area of COP-SO_3_H was calculated as 44.77 m²·g^−1^, and the pore size distribution was primarily concentrated at approximately 1.25 nm and 3.5–7 nm, indicating that COP-SO_3_H is a porous material containing both micro- and meso-pores. Furthermore, the thermal and chemical stabilities of COP-SO_3_H were examined via thermogravimetric analysis (TGA) under a nitrogen atmosphere. The TGA results (Figure 4G) indicate that COP-SO_3_H maintained stability up to 336.1 °C. Moreover, immersing COP-SO_3_H in HCl (3 M) and NaOH (3 M) solutions revealed that HCl@COP-SO_3_H and NaOH@COP-SO_3_H exhibited XRD patterns (Figure 4H) consistent with those of pristine COP-SO_3_H, revealing no new diffraction peaks. This result aligns with the amorphous nature of COP-SO_3_H and confirms its high stability under extreme conditions.

### 2.2. Batch-Wise Adsorption Experiments

#### 2.2.1. Effect of Adsorbent Dosage on Adsorption Performance

**Figure 5:**
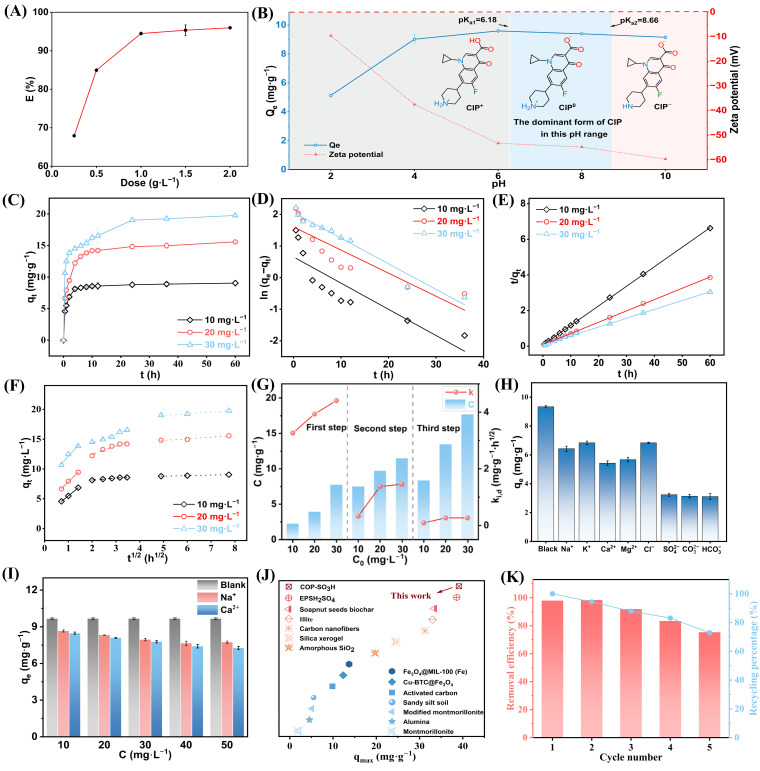
(**A**) Impact of dosage on CIP adsorption onto COP-SO_3_H. (**B**) Influence of pH on CIP adsorption and zeta potential of COP-SO_3_H. (**C**) Kinetic study of CIP adsorption onto COP-SO_3_H at 10, 20, and 30 mg·L^−1^ initial concentrations. (**D**) Pseudo-first-order kinetics of COP-SO_3_H for CIP adsorption at different concentrations. (**E**) Pseudo-second-order kinetics of COP-SO_3_H for CIP adsorption at different concentrations. (**F**) Intra-particle diffusion model for CIP adsorption onto COP-SO_3_H. (**G**) Different intra-particle diffusion model parameters (C and K_i,d_) with concentration. (**H**) Effect of various inorganic ions on CIP adsorption onto COP-SO_3_H. (**I**) Influence of ionic strength on CIP adsorption onto COP-SO_3_H. (**J**) Comparison of the maximum adsorption capacities for CIP. (**K**) Results of five continuous cycles of reusing COP-SO_3_H for CIP adsorption.

The influence of adsorbent dosage on the removal efficiency of CIP was further assessed to optimize the COP-SO_3_H dosage for the experiments, aiming to maintain cost-effectiveness and pollutant removal efficacy. The COP-SO_3_H loading ratio (ratio of material mass to CIP solution volume) was varied across five gradients, ranging from 0.25 g·L^−1^ to 2 g·L^−1^, for the adsorption experiments. The aforementioned material was added to a 50 mL centrifuge tube, followed by 20 mL of pollutant solution at a concentration of 10 mg·L^−1^. Subsequently, the mixture was placed on an open-air orbital shaker and agitated for 24 h. Subsequently, the concentration of FQ was determined. The results shown in Figure 5A indicate that, as the adsorbent dosage increased, the removal efficiency gradually increased from 67.92% to 95.97%. This trend can be attributed to the increase in the effective surface area and adsorption sites of the material for pollutants [35]. However, because the amount of pollutants in the system remains fixed, excessive addition of adsorbent material results in the aggregation of adsorbent particles [36]. This leads to unoccupied adsorption sites, where pollutants remain uncaptured, rendering an increased dosage of adsorbent material only marginally effective in enhancing the removal efficiency of CIP [37]. Consequently, the CIP removal efficiency slowly increased when the dosage was between 1 and 2 g·L^−1^. Considering the removal efficiency and experimental cost, the optimal COP-SO_3_H dosage was 1.0 g·L^−1^.

#### 2.2.2. Effect of pH on Adsorption Performance

During operation, wastewater treatment plants must treat sewage at various pH levels. Therefore, the adsorbent must efficiently capture antibiotics over a wide pH range, making the pH value of wastewater a significant reference factor influencing the adsorption process. Figure 5B shows the impact of pH (ranging from 2.0 to 10.0) on the adsorption capacity of COP-SO_3_H for CIP. At an initial CIP concentration of 10 mg·L^−1^ and a temperature of 293 K, the equilibrium adsorption capacity (q_e_) of CIP on COP-SO_3_H increased from 5.1 mg·L^−1^ to 9.6 mg·L^−1^ as the pH increased, and then slightly decreased to 9.15 mg·L^−1^. Notably, CIP exists in three forms, depending on the pH: cationic (CIP^+^) at pH < 6.18, anionic (CIP^−^) at pH > 8.15, and zwitterionic (CIP^0^) at 6.18 < pH < 8.15. The zeta potential of COP-SO_3_H was negatively charged and increased with increasing pH. Based on the experimental results, as the pH increased from 4 to 6, the negative charge of COP-SO_3_H significantly strengthened, increasing the adsorption capacity. As the pH approached neutrality, the strong hydrophobic interaction between CIP^0^, CIP^±^, and COP-SO_3_H, coupled with enhanced π–π electron donor–acceptor (EDA) interactions due to electrostatic attraction, significantly increased the adsorption affinity between them. Conversely, strong electrostatic repulsion weakened the π–π EDA interaction, thereby inhibiting the adsorption effectiveness of COP-SO_3_H for CIP. Therefore, the influence of pH on the CIP adsorption capacity of COP-SO_3_H depends on the balance between the electrostatic, hydrophobic, and π-π EDA interactions. Because q_e_ peaked at pH 6, this pH level was selected as the most suitable for our experiments.

#### 2.2.3. Influence of Contact Time on Adsorption/Adsorption Kinetics

To evaluate the impact of contact time on adsorption, 1 g∙L^−1^ CIP solutions with initial concentrations of 10, 20, and 30 mg·L^−1^ were exposed to COP-SO_3_H for varying durations. The temperature was maintained at 20 °C. Figure 5C shows that the equilibrium time for CIP adsorption onto COP-SO_3_H is within 24 h for all three initial concentrations. The adsorption capacities of COP-SO_3_H for CIP with initial concentrations of 10, 20, and 30 mg·L^−1^ were 9.05, 15.57, and 19.76 mg·L^−1^, respectively. These results indicated that increasing the initial CIP concentration enhanced the adsorption capacity.

Three kinetic models were fitted to the data to understand the adsorption process and mechanism: pseudo-first-order, pseudo-second-order, and intra-particle diffusion models. The equations used are as follows [38,39,40]:

(1) $$\frac{dq_{t}}{dt}=k_{1}(q_{e}-q_{t})$$

(2) $$n(q_{e}-q_{t})=\ln q_{e}-k_{1}t$$

(3) $$\frac{dq_{t}}{dt}=k_{2}(q_{e}-q_{t}{)}^{2}$$

(4) $$\frac{t}{q_{t}}=\frac{1}{q_{e}}t+\frac{1}{k_{2}q_{e}^{2}}$$

(5) $$q_{t}=k_{i,d}t^{1/2}+C_{i}$$

where q_e_ (mg) and q_t_ (mg) represent the adsorption amounts of CIP on COP-SO_3_H at equilibrium and time t, respectively, k_1_ (h^−1^) is the first-order adsorption rate constant, k_2_ (g·mg^−1^·h^−1^) is the second-order adsorption rate constant, and C_i_ (mg·g^−1^) is the parameter representing the thickness of the adsorbed CIP layer.

Based on the fitting results (Figure 5D,E and Table 2), the pseudo-second-order model demonstrated a stronger fit (R^2^ > 0.99) than the pseudo-first-order model. Additionally, the experimentally determined adsorption capacities (q_e,exp_) closely aligned with the calculated values (q_e,cal_) from the pseudo-second-order model, indicating that it describes the adsorption process of CIP onto COP-SO_3_H more accurately than the pseudo-first-order kinetic model. These results underscore that chemical adsorption is the primary mechanism governing CIP adsorption onto COP-SO_3_H.

Intra-particle diffusion analysis (Figure 5F and Table 3) revealed a three-stage diffusion process of CIP within COP-SO_3_H. The initial stage is the external diffusion phase, where the adsorption rate k_i,1_ reached its maximum at the same concentration, indicating rapid adsorption. This suggests that CIP molecules migrated from the solution to the external surface of COP-SO_3_H during initial adsorption. The second stage is the internal diffusion phase, with the adsorption rate k_i,1_ > k_i,2_, indicating gradual adsorption. This suggests that the CIP molecules saturated the external surface of COP-SO_3_H and diffused from the outer surface to the inner surface of the material. The third stage is the desorption–adsorption equilibrium phase, where the adsorption rate k_i,2_ > k_i,3_ ≈ 0. This indicated that the CIP molecules reached saturation on both the external and internal surfaces of COP-SO_3_H, and the desorption and adsorption of CIP molecules reached equilibrium. However, none of the diffusion curves passed through the origin, suggesting that the entire adsorption process was influenced by multiple steps [41].

#### 2.2.4. Adsorption Isotherms and Thermodynamics

**Figure 6:**
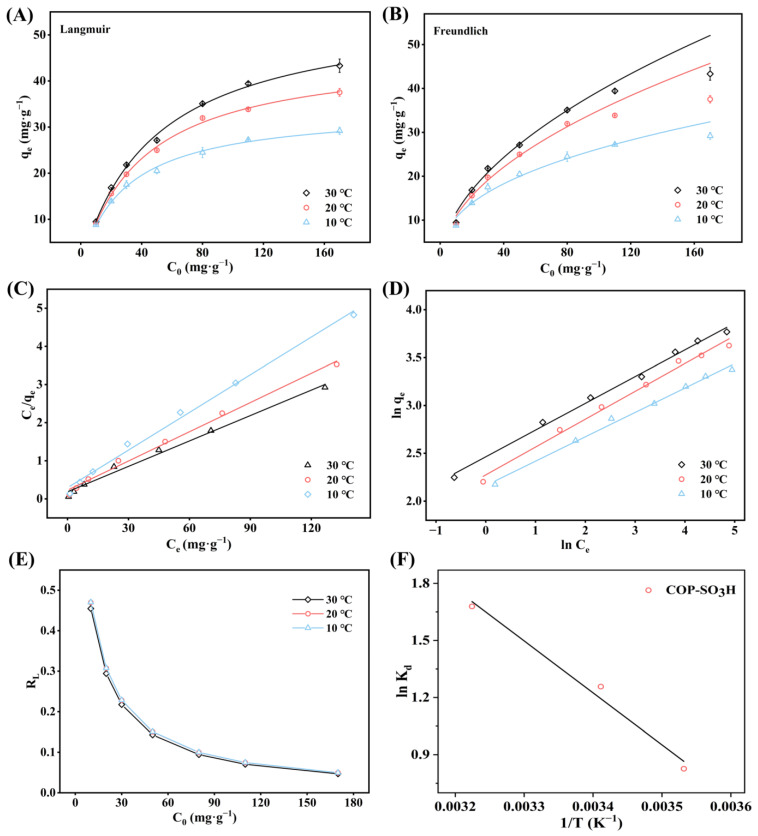
(**A**,**B**) Adsorption isotherms of COP-SO_3_H for CIP at 10, 20, and 30 °C. (**C**) Langmuir linear adsorption fit of COP-SO_3_H for CIP at 10, 20, and 30 °C. (**D**) Freundlich linear adsorption fit of COP-SO_3_H at 10, 20, and 30 °C. (**E**) Relationship between R_L_ and initial concentration. (**F**) Thermodynamic fit of CIP adsorption onto COP-SO_3_H.

This section explores the maximum CIP adsorption capacity of COP-SO_3_H and its interactions. The adsorption results at CIP concentrations ranging from 10 to 170 mg·L^−1^ under three different temperature conditions (10, 20, and 30 °C) were investigated. The experimental data were fitted using single-layer Langmuir and multi-layer Freundlich adsorption isotherm models (Figure 6A,B), as represented by Equations (6) to (8) [42,43]:

(6) $$\frac{C_{e}}{q_{e}}=\frac{1}{K_{L}q_{m}}+\frac{C_{e}}{q_{m}}$$

(7) $$R_{L}=\frac{1}{1+K_{L}C_{0}}$$

(8) $$q_{e}=K_{F}C_{e}^{1/n}$$

where q_e_ (mg·g^−1^) represents the adsorption capacity of the adsorbent for the adsorbate CIP at adsorption equilibrium, C_e_ (mg·g^−1^) is the residual concentration of CIP in the solution at adsorption equilibrium, q_m_ (mg·g^−1^) denotes the theoretical maximum adsorption capacity per unit mass of adsorbent COP-SO_3_H, K_L_ (L·mg^−1^) is the Langmuir equilibrium constant, 1/n represents the adsorption intensity, and R_L_ indicates the difficulty level of the adsorption process.

The results indicated a significant increase in the adsorption capacity of COP-SO_3_H for CIP with higher initial concentrations under the same temperature conditions. This trend suggests that elevated initial concentrations promote CIP adsorption onto COP-SO_3_H by creating a larger concentration difference, thereby acting as a pivotal driving force for adsorption [44,45]. This reduction in the mass transfer resistance between CIP in the solution and COP-SO_3_H enhanced the overall efficiency. As shown in Table 4, the Langmuir model exhibited a better fit (R^2^ > 0.99) than the Freundlich model, indicating that the single-layer adsorption mechanism better described the CIP adsorption process of COP-SO_3_H. According to the Langmuir model, the theoretical maximum adsorption capacities of COP-SO_3_H for CIP at 10, 20, and 30 °C were 30.37, 39.11, and 44.96 mg·g^−1^, respectively. Importantly, COP-SO_3_H exhibited superior adsorption performance compared to other adsorbents, such as modified montmorillonite, sandy silt soil, and carbon nanofibers (Figure 5J and Table 5), highlighting its value as a practical adsorbent. Furthermore, the calculated R_L_ values for CIP adsorption onto COP-SO_3_H at 10, 20, and 30 °C ranged from 0.047 to 0.470, falling between 0 and 1. This suggests that the adsorption equilibrium between COP-SO_3_H and CIP promotes the adsorption process effectively.

Adsorption thermodynamics helps to understand the effect of temperature on the adsorption process and can further elucidate the adsorption mechanism of CIP onto COP-SO_3_H. At different temperatures, the values of Gibbs’ free energy change (ΔG^0^, kJ·mol^−1^), entropy change (ΔH^0^, kJ·mol^−1^), and enthalpy change (ΔS^0^, kJ·mol^−1^·K^−1^) were calculated using Equations (9)–(11) as follows [55]:

(9) $$\Delta G^{0}=-RTln\;K_{a}$$

(10) $$K_{a}=10^{6}K_{L}$$

(11) $$\ln K_{a}=\frac{\Delta S^{\circ }}{R}-\frac{\Delta H^{\circ }}{RT}$$

where R is the universal gas constant (8.3145 J·mol^−1^·K^−1^), and T is the solution temperature (K). K_a_ is the thermodynamic equilibrium constant without units, which can be obtained by multiplying Langmuir equilibrium constant K_L_ (L·mg^−1^) by 10^6^. As shown in Table 6, the values of ΔG^0^ were negative at 10, 20, and 30 °C, indicating that the adsorption of CIP onto COP-SO_3_H was spontaneous. With increasing temperature, the values of ΔG^0^ decreased from −1.95 to −4.23 kJ·mol^−1^, indicating that higher temperatures enhanced the adsorption performance of COP-SO_3_H. This observation was consistent with the Langmuir model fitting results. Moreover, the obtained value of ΔH^0^ suggested that the adsorption of CIP onto SO_3_H was an endothermic process.

#### 2.2.5. Influence of Inorganic Ion Type and Ionic Strength on Adsorption

To assess the impact of inorganic ions in the wastewater on the adsorption of CIP onto COP-SO_3_H, eight ions were selected for experimentation: Na^+^, K^+^, Ca^2+^, and Mg^2+^ (as cations) and Cl^−^, SO_4_^2−^, CO_3_^2−^, and HCO_3_^−^ (as anions). The effects of different ion concentrations (10 mM) on CIP adsorption onto COP-SO_3_H were also investigated. Notably, the hydrogen bonds between the oxygen acid ions and COP-SO_3_H enhanced the affinity between these ions and CIP. Consequently, oxygen-containing acid ions suppressed the adsorption of CIP onto COP-SO_3_H. Furthermore, the effects of the Na^+^ and Ca^2+^ concentrations on adsorption were examined (Figure 5I). As the Na^+^ and Ca^2+^ concentrations increased, the hydrophobic interactions between COP-SO_3_H and CIP intensified. Simultaneously, the competition between CIP and Na^+^ or Ca^2+^ resulted in a substantial initial decrease, followed by a plateau in the adsorption amount of COP-SO_3_H for CIP.

#### 2.2.6. Recyclability

**Figure 7:**
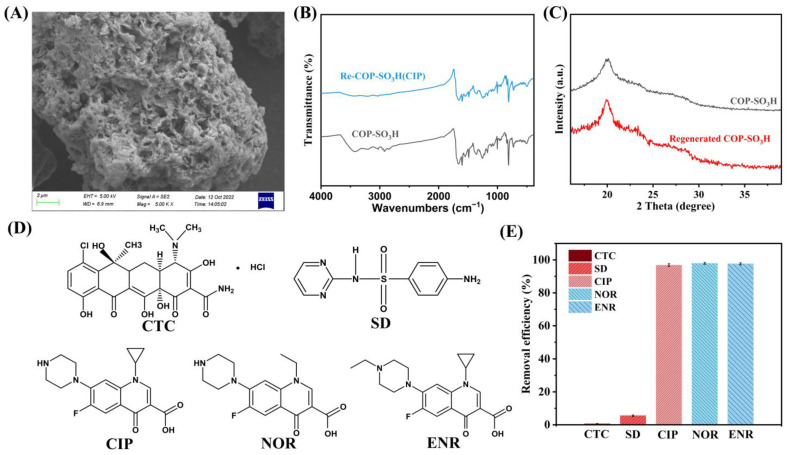
Regeneration of COP-SO_3_H after five cycles of reuse: (**A**) SEM, (**B**) FT-IR spectra, and (**C**) XRD. (**D**) Structures of CTC, SD, CIP, NOR, and ENR. (**E**) Removal efficiency of COP-SO_3_H for CTC, SD, CIP, NOR, and ENR.

The recyclability of adsorbents has economic value for wastewater treatment. Highly recyclable adsorbents contribute to both stability and cost reduction during practical use. Methanol was employed as a desorption agent for five consecutive adsorption–desorption cycles of CIP onto COP-SO_3_H. Based on the results, the CIP removal efficiency of COP-SO_3_H and its recovery efficiency in each cycle were investigated. The results indicated that the removal efficiency could reach 92% by the third cycle, and with an increase in the number of cycles to five, the removal efficiency of COP-SO_3_H for CIP still remained at 75% (Figure 5K). SEM, FT-IR, and XRD analyses (Figure 7A–C) revealed no significant structural changes in COP-SO_3_H after five cycles. This finding demonstrates that COP-SO_3_H is a reusable adsorbent.

#### 2.2.7. Adsorption Selectivity

The applicability of a material to water treatment depends on its adsorption selectivity. To investigate the adsorption selectivity of COP-SO_3_H, its efficiency in removing sulfonamide, tetracycline, and quinolone antibiotics was assessed at a pH of 6. Five antibiotics were selected, i.e., tetracycline (chlorotetracycline, CTC), sulfonamide (sulfadiazine, SD), and quinolones (ciprofloxacin, CIP; norfloxacin, NOR; and enrofloxacin, ENR). The structural formulae are shown in Figure 7D. When COP-SO_3_H was used to adsorb each antibiotic, the removal efficiencies were 0.52%, 5.50%, 96.83%, 97.87%, and 97.61%, respectively (Figure 7E). COP-SO_3_H exhibited a favorable adsorption performance for CIP, NOR, and ENR because of their similar chemical structures. The electrostatic interactions between Form-II of COP-SO_3_H and quinolone antibiotics (FQs) resulted in a stronger affinity of COP-SO_3_H for FQs than for other antibiotics. Consequently, the adsorption sites on the COP-SO_3_H adsorbent exhibited selective binding capability for FQs.

## 3. Synthesis of COP-SO_3_H

The porous structures are crucial for adsorption. The existence of pores serves as a precondition for various interactions and is a significant factor influencing the performance of porous adsorbents [56]. COP-SO_3_H, with its microporous and mesoporous channel structures, can capture CIP. To explore the possibility of CIP entering the pores during adsorption, the adsorbed material (CIP@COP-SO_3_H) underwent N_2_ adsorption–desorption tests at 160 °C for 12 h. After adsorption, the BET surface area was reduced to 26.73 m²·g^−1^, indicating a 40% decrease, confirming CIP adsorption within the pores, which is known as the pore-filling effect.

According to the effects of the pH and various ions on the adsorption capacity of COP-SO_3_H for CIP, as well as the zeta potential analysis, at a pH of 6, the zeta potential of CIP@COP-SO_3_H (9.3 mV) was significantly higher than that of CIP@COP-SO_3_H (9.3 mV), indicating that electrostatic interactions occurred during adsorption. Furthermore, analyzing the XPS profiles before and after COP-SO_3_H adsorbed CIP revealed that the peaks in the N 1s spectrum after adsorption shifted from binding energies of 400.5 and 399.8 eV to 400.6 and 399.6 eV, respectively. This suggests that electrostatic interactions occur between COP-SO_3_H and CIP.

**Figure 8:**
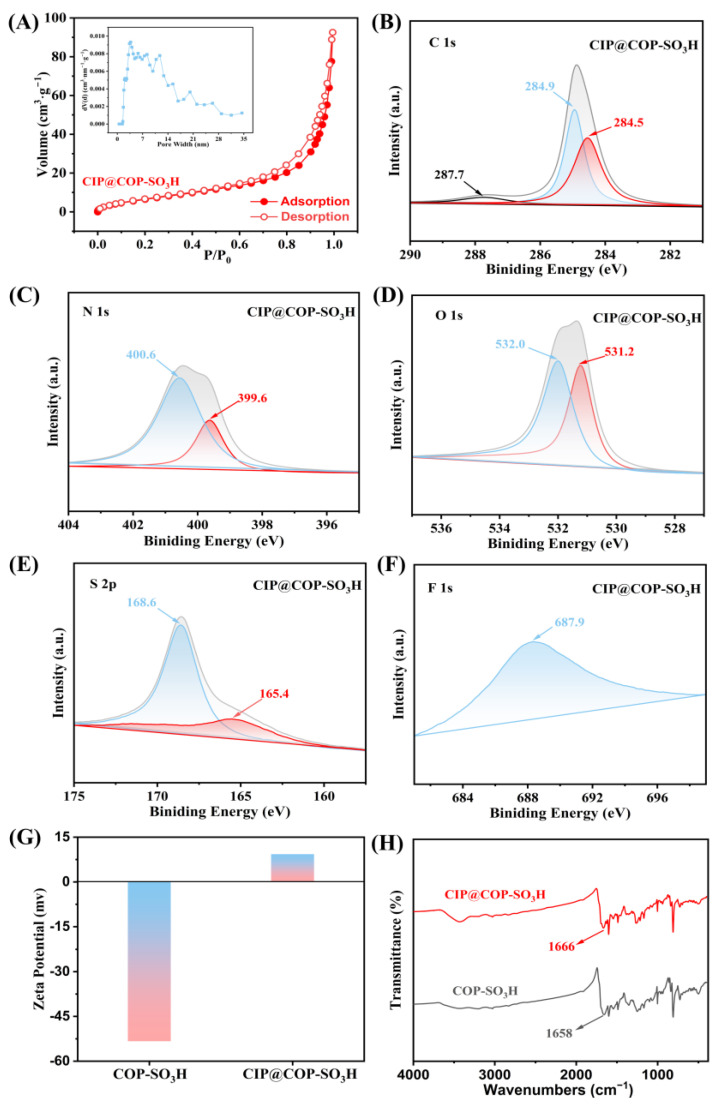
(**A**) Adsorption–desorption curves and pore size distribution of CIP@COP-SO_3_H. (**B**–**F**) XPS spectra of C 1s, N1s, O1s, S2p, and F1s of CIP@COP-SO_3_H. (**G**) Zeta potential values for COP-SO_3_H and CIP@COP-SO_3_H. (**H**) Infrared spectra of COP-SO_3_H and CIP@COP-SO_3_H.

Even when subjected to weak electrostatic interactions between COP-SO_3_H and CIP or strong electrostatic repulsions, the adsorption performance remained adequate. We speculate that other factors also affect the adsorption process. Notably, in the C 1s spectrum of COP-SO_3_H, the peaks corresponding to the C=O bond (288.2 eV), C–S/C=N bond (258.0 eV), and C–C/C=C bond (284.4 eV) shifted to 287.7, 284.9, and 284.5 eV, respectively (Figure 8B). This can be attributed to the π-π EDA interactions between the electron-rich framework of COP-SO_3_H and CIP molecules, which led to the capture of CIP. Additionally, a new peak at 687.9 eV in the CIP@COP-SO_3_H spectrum indicates that COP-SO_3_H may have captured the F element present in the CIP molecule.

Further analysis of the infrared spectra before and after the adsorption by COP-SO_3_H (Figure 8H) revealed that the adsorption peak at 1658 cm^−1^, associated with the C=O bond, shifted to 1666 cm^−1^. This indicated hydrogen bonding interactions between the COP-SO_3_H material and CIP. Specifically, the -SO_3_H and -CO-NH- functional groups anchored onto the surface of the material demonstrated the capability to form hydrogen bonds with the -NH_2_, -COOH, and -F functional groups present within the CIP structure.

Apart from the four interactions mentioned above, the hydrophilic–lipophilic balance in the adsorption process cannot be overlooked. Both the COP-SO_3_H and CIP molecules contained several hydrophilic and hydrophobic groups. For instance, the carboxyl and hydroxyl groups in COP-SO_3_H play a hydrophilic role when binding to CIP molecules, increasing the speed at which CIP enters the pores. Additionally, both the COP-SO_3_H material and CIP molecules contained aromatic rings, which promoted hydrophobic interactions between the two. These two effects form a hydrophilic–lipophilic balance during adsorption [57].

**Figure 9:**
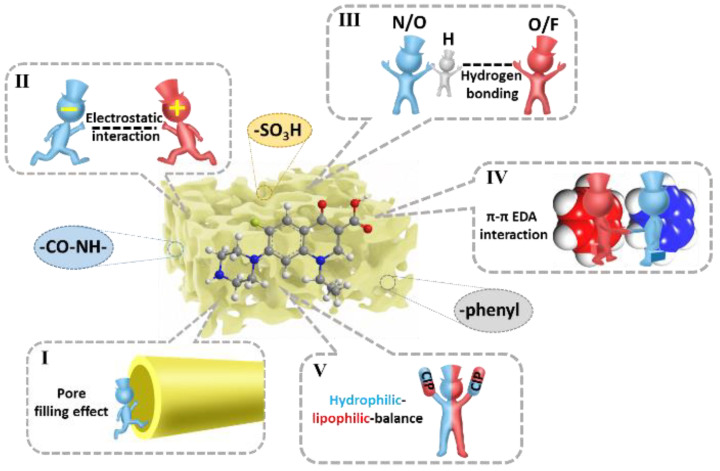
Mechanism of CIP adsorption by COP-SO_3_H.(I: the pore filling effect in polymers, II: the electrostatic interactions in the guest molecules and the framework, III: the formation of hydrogen bonding between adsorbent and adsorbate, IV: the π-π EDA interactions between CIP and COP-SO_3_H, V: the Hydrophilic–lipophilic balance between the target pollutant and COP-SO_3_H).

In summary, a plausible mechanism for CIP adsorption on COP-SO_3_H was inferred. This mechanism involves a combination of the pore-filling effect, electrostatic interactions, hydrogen bonding, π-π EDA interactions, and hydrophilic–lipophilic balance, all of which collectively determine the adsorption efficiency of the material (as illustrated in Figure 9).

## 4. Experimental Materials and Methodology

### 4.1. Experimental Materials

Benzene-1,3,5-tricarbohydrazide (BTCH) was prepared in the laboratory using previously reported methods [58], and 4,4”-p-Terphenyldicarboxaldehyde (TPDA) and 2,5-diaminobenzenesulfonic acid (DABA) were sourced from Sinopharm Chemical Reagent Co. Ltd. (Shanghai, China).

### 4.2. COP-SO_3_H Characterization

The microstructures of COP-SO_3_H were observed using scanning electron microscopy (SEM, ZEISS, Gemini Sigma 300, Oberkochen, Germany) and transmission electron microscope (TEM, FEI, Tecnai F20, Hillsboro, OR, USA). The morphological changes in the raw material COP-SO_3_H and the COP-SO_3_H material after adsorption and subsequent cleaning were examined using a Zeiss Sigma 300 microscope. Thermogravimetric analysis of COP-SO_3_H was conducted using a NETZSCH STA 2500 thermal analyzer (Free State of Bavaria, Germany) at a heating rate of 10 °C·min^−1^ under a protective N_2_ atmosphere. XRD spectra in the 4–40° range were acquired using a powder X-ray diffractometer (ESCALAB 250Xi, Thermo Fisher Scientific, Waltham, MA, USA). Solid-state 13C cross-polarization/magic angle spinning nuclear magnetic resonance (CP/MAS NMR) spectra were recorded at 5 kHz. Elemental analyses of C,H,N, and S were conducted using an Elementar Vario Micro analyzer. The FT-IR of COP-SO_3_H, which was pressed into KBr powder, was performed within the range of 400–4000 cm^−1^ using a Nexus 410 infrared spectrometer. X-ray photoelectron spectroscopy (XPS) was performed using an ESCALAB 250Xi instrument. The N_2_-specific surface area of COP-SO_3_H was measured using an Autosorb iQ2 instrument (QuantaChrome Instruments Corp., Florida, USA). Additionally, the point of zero charge of COP-SO_3_H at different pH values was determined by the pH drift method using a zeta potential meter (Zetasizer Nano ZS90, Malvern Panalytical, Malvern, UK). Furthermore, the initial and equilibrium concentrations of CIP were measured at a wavelength of 271 nm using a Shimadzu UV-2550 spectrophotometer.

### 4.3. Synthesis of COP-SO_3_H

BTCH (0.2 mmol, 50.4 mg), TPDA (0.6 mmol, 171.8 mg), and DABA (0.3 mmol, 56.5 mg) were weighed and added to a headspace vial containing dimethyl sulfoxide (DMSO, 5 mL) as a solvent. The three compounds were thoroughly mixed, heated to 100 °C, and maintained for 15 min, to yield a red polymer. The polymer was transferred to a dialysis bag and dialyzed with distilled water to remove the organic solvent. After dialysis, the material was transferred to a conical flask and freeze-dried. The resulting pale-red powder was COP-SO_3_H.

### 4.4. Adsorption Experiments

For all the experiments, CIP was dissolved in distilled water to obtain the required solution. In each instance, COP-SO_3_H was mixed with an antibiotic in a 50 mL plastic centrifuge tube and agitated in a gas bath shaker at 150 r·min^−1^. Periodically, a specific volume of supernatant was extracted via centrifugation. The remaining CIP concentration was determined via ultraviolet–visible spectrophotometry at λ = 271 nm. Different amounts of COP-SO_3_H (0.25, 0.5, 1, 1.5, and 2 g·L^−1^) were mixed in a 20 mL CIP solution with an initial concentration of 10 mg·L^−1^ to examine the effects of adsorbent dosage on the adsorption properties of COP-SO_3_H. The effect of the initial pH on the adsorption of CIP by COP-SO_3_H was investigated at an initial CIP concentration of 10 mg·L^−1^, COP-SO_3_H addition of 1 g·L^−1^, and pH values ranging from 2 to 10. At 20 °C, adsorption kinetics experiments were performed by mixing 1 g·L^−1^ of COP-SO_3_H with a CIP solution (40 mL volume, pH = 6, initial concentrations of 10, 20, and 30 mg·L^−1^) in a 50 mL centrifuge tube. Intermittent adsorption experiments were conducted at 10, 20, and 30 °C by mixing 1 g·L^−1^ COP-SO_3_H with 40 mL of CIP (concentrations of 10, 20, 30, 50, 80, 110, and 170 mg·L^−1^) in a 50 mL centrifuge tube. This was performed to explore the isothermal adsorption characteristics of CIP on COP-SO_3_H. Using a CIP solution without added ions as a control, the influence of ion competition on the adsorption of CIP by COP-SO_3_H was studied by adding ions, such as Na^+^, K^+^, Ca^2+^, Mg^2+^, Cl^−^, CO_3_^2−^, HCO_3_^−^, and SO_4_^2−^ at a concentration of 10 mM. Additionally, the effects of different concentrations of Na^+^ and Ca^2+^ (ranging from 0 to 50 mg·L^−1^) on the adsorption of CIP by COP-SO_3_H were explored. The adsorption quantity of the COP-SO_3_H material at time t, denoted as q_t_ (mg·L^−1^), equilibrium adsorption quantity, denoted as q_e_ (mg·L^−1^), and removal efficiency (%) were determined using the following formulas [59,60]:

(12) $$q_{t}=\frac{V(C_{0}-C_{t})}{m}$$

(13) $$q_{e}=\frac{V(C_{0}-C_{e})}{m}$$

(14) $$E\left(\%\right)=\frac{C_{0}-C_{e}}{C_{0}}\times 100\%$$

where C_0_(mg·L^−1^) is the initial concentration of CIP; C_e_ (mg·L^−1^) and C_t_ (mg·L^−1^) are the concentrations of CIP at equilibrium and at adsorption time t, respectively; m (g) represents the mass of COP-SO_3_H added; and V (L) is the volume of the CIP solution.

## 5. Conclusions

In summary, based on the three monomers, BTCH, TPDA, and DABA, a ternary covalent organic polymer (COP-SO_3_H) anchored with -SO_3_H was designed using the Schiff reaction and a multicomponent solvothermal method. This design endowed the polymer with porous structural characteristics, abundant π-conjugated phenyl rings, and abundant CO-NH and SO_3_H functional groups, which facilitated the removal of CIP from water. The experimental results showed that COP-SO_3_H achieved a good adsorption performance over a wide pH range of 4–10. The adsorption process followed pseudo-second-order kinetics and followed the Langmuir model. Moreover, BET, XPS, FT-IR, and zeta potential analyses indicated that the adsorption mechanism involved pore-filling effects, electrostatic interactions, hydrogen bonding, π-π EDA interactions, and the hydrophilic–lipophilic balance. After five cycles, the material maintained a removal efficiency of >75%, confirming the reusability of COP-SO_3_H. The present study investigates the adsorption behavior of COP-SO_3_H on FQs (using CIP as a representative) in aqueous environments, thereby expanding the application potential of designed functionalized COPs for selective pollutant removal and water environment remediation.

## Figures and Tables

**Table 1:** Elemental analysis of COP-SO_3_H.

Materials	C (wt %)		H (wt %)		N (wt %)		S * (wt %)	
	Calc.	Exp.	Calc.	Exp.	Calc.	Exp.	Calc.	Exp.
COP-SO_3_H	66.92	74.17	3.85	4.91	10.76	6.58	6.15	0.62

* Instrument error results in discrepancy in the measurement of S content.

**Table 2:** Kinetic parameters for CIP adsorption by COP-SO_3_H.

C_0_	q_e,exp_	Removal	Pseudo-First-Order Dynamics Model				Pseudo-Second-Order Dynamics Model			
(mg·L^−1^)	(mg·g^−1^)	Efficiency	q_e,cal_	k_1_	Δq_1_	R^2^	q_e,cal_	k_1_	Δq_1_	R^2^
		(%)	(mg·g^−1^)	(h^−1^)	(%)		(mg·g^−1^)	(h^−1^)	(%)	
10	9.05	90.50	1.92	0.082	370.3	0.71	9.11	0.170	0.65	0.99
20	15.57	77.85	4.96	0.073	214.0	0.74	15.73	0.057	1.01	0.99
30	19.76	65.87	7.83	0.081	152.3	0.96	20.12	0.033	1.79	0.99

**Table 3:** Intra-particle diffusion model parameters for CIP adsorption by COP-SO_3_H.

C_0_	Intraparticle Diffusion Model								
(mg·L^−1^)	k_i,1_	C_1_	R^2^	k_i,2_	C_2_	R^2^	k_i,3_	C_3_	R^2^
	(mg·g^−1^·h^−1/2^)	(mg·g^−1^)		(mg g^−1^ h^−1/2^)	(mg·g^−1^)		(mg·g^−1^·h^−1/2^)	(mg·g^−1^)	
10	3.26	2.25	0.99	0.33	7.50	0.96	0.09	8.35	0.99
20	3.94	3.92	0.99	1.37	9.72	0.89	0.27	13.47	0.91
30	4.41	7.74	0.99	1.46	11.48	0.96	0.27	17.68	0.97

**Table 4:** Adsorption parameters of isotherm models for the CIP adsorption by COP-SO_3_H.

T	Langmuir Isotherm			Freundlich Isotherm		
(°C)	q_m_	K_L_	R^2^	K_F_	n	R^2^
	(mg·g^−1^)	(L·mg^−1^)		(mg·g^−1^) (L·mg^−1^)^1/n^		
10	30.37	0.11	0.99	8.70	3.93	0.99
20	39.11	0.11	0.99	9.73	3.44	0.98
30	44.96	0.11	0.99	11.75	3.58	0.98

**Table 5:** Comparison of the maximum adsorption capacity of CIP by different adsorbents.

S.N.	Adsorbents	Adsorption Capacity (mg·g^−1^)	Conditions	Reference
1	Montmorillonite	1.94	298 K	[46]
2	Alumina	4.55	298 K	[46]
3	Modified montmorillonite	5.10	298 K	[46]
4	Sandy silt soil	5.50	298 K	[47]
5	Activated carbon	9.87	298 K	[46]
6	Cu-BTC@Fe_3_O_4_	12.35	298 K	[48]
7	Fe_3_O_4_@MIL-100 (Fe)	13.65	298 K	[49]
8	Amorphous SiO_2_	19.71	298 K	[38]
9	Silica xerogel	24.45	298 K	[50]
10	Carbon nanofibers	31.26	298 K	[51]
11	Illite	33	/	[52]
12	Soapnut seeds biochar	33.44	303 K	[53]
13	EPS_H2SO4_	38.61	/	[54]
14	COP-SO_3_H	39.11	293 K	This work

**Table 6:** Thermodynamic parameters for the CIP adsorption by COP-SO_3_H.

Temperature (K)	∆G^0^ (kJ·mol^−1^)	∆H^0^ (kJ·mol^−1^)	∆S^0^ (kJ·mol^−1^·K^−1^)
283	−1.95	22.69	0.08734
293	−3.06		
303	−4.23		

## Data Availability

Available on demand.
